# Serum Aquaporin-4 Antibody Status and TGF-β in Neuromyelitis Optica Spectrum Disorder: Impact on Astrocyte Function and Correlation with Disease Activity and Severity

**DOI:** 10.3390/neurolint17120200

**Published:** 2025-12-09

**Authors:** Vinicius Gabriel Coutinho-Costa, Isadora Matias, Renan Amphilophio Fernandes, Michele Siqueira, Larissa Araujo Duarte, Beatriz Martins Fernandes, Jorge Marcondes de Souza, Soniza Vieira Alves-Leon, Flávia Carvalho Alcantara Gomes

**Affiliations:** 1 Instituto de Ciências Biomédicas, Universidade Federal do Rio de Janeiro, Rio de Janeiro 21941-902, Brazil; 2 Hospital Universitário Clementino Fraga Filho, Universidade Federal do Rio de Janeiro, Rio de Janeiro 21941-913, Brazil; 3 Instituto Biomédico, Universidade Federal do Estado do Rio de Janeiro, Rio de Janeiro 20211-030, Brazil; 4 Faculdade de Medicina, Universidade Federal do Rio de Janeiro, Rio de Janeiro 21941-901, Brazil

**Keywords:** NMOSD, biomarker, astrocytes, cytokines, demyelinating diseases, AQP4, TGF-β

## Abstract

Background: Neuromyelitis optica spectrum disorder (NMOSD) involves demyelinating astrocytopathy. Most cases have autoantibodies against aquaporin-4 (AQP4 ab), but AQP4 ab-negative patients may also meet NMOSD criteria. Overlapping clinical phenotypes of CNS inflammatory demyelinating diseases (IDDs) complicate understanding NMOSD mechanisms. Objectives: Investigate molecules related to neuroinflammation and astrocyte function as potential biomarkers of NMOSD and other IDDs by using clinical data and in vitro assays. Methods: Subjects (176) with different IDDs (NMOSD (37), MS (125), MOGAD (3), ADEM (3) and eight radiologic isolated syndromes (RIS)) were studied. Plasma concentrations of TGF-β and other cytokines were measured by single molecule array (SIMOA), Luminex and ELISA assays. Functional assays used in vitro cultured human astrocytes exposed to NMOSD subjects’ serum, followed by immunolabeling. Results: TGF-β levels were higher in NMOSD patients during attacks compared to inactive phases. AQP4+ groups in inactive phases had lower TGF-β levels than AQP4− groups. No significant difference was found for IL-1β, IL-8, IL-10, IL-17A and Thrombospondin plasma concentrations, with a minor difference for VEGF in the AQP4+ group. Astrocytes exposed to NMOSD AQP4+ and AQP4− subjects serum, with or without TGF-β1, showed no changes in C3, NFkB and HMGB1. However, the content of GLT-1 decreased in AQP4+ serum-treated astrocytes, reversed by TGF-β1. Conclusions: TGF-β may be a potential NMOSD activity biomarker, indicating different disease mechanisms based on AQP4 ab presence.

## 1. Introduction

Inflammatory demyelinating diseases (IDDs) of the central nervous system (CNS) represent a prevailing cause of early neurological disability. They are a burden on patient’s quality of life and both government and individual costs [1]. While the prototypical IDD is multiple sclerosis (MS), those include diseases such as neuromyelitis optica spectrum disorder (NMOSD), myelin oligodendrocyte glycoprotein (MOG) antibody-associated disease (MOGAD), acute disseminated encephalomyelitis (ADEM) and other rarer forms of CNS demyelination.

Early differentiation among CNS IDDs may be difficult due to the clinical overlap among their manifestations. Diagnosis depends on imaging and biomarker testing, which are costly and of limited availability in several countries. Recently, the use of molecules correlated to neuronal (neurofilament light chain; NfL) and glial (glial fibrillary acid protein; GFAP) injury as a proxy to monitor CNS lesion has been established in clinical practice [2].

Recent decades have shown great advancements in early detection and treatment of CNS IDDs. One of them was the discovery of the pathogenic aquaporin-4 autoantibody (AQP4 ab) in NMOSD patients [3]. Nevertheless, there is a fraction of clinically defined NMOSD patients who do not present the autoantibody [4]. AQP4 is a water channel enriched in the CNS, where it is mainly responsible for water homeostasis, mostly located at the astrocyte endfeet [5]. Therefore, NMOSD has been considered an astrocytopathy [6]. This may not be the case for AQP4 ab− disease, where disease targets are poorly understood. In contrast, AQP4 ab patients have complement system activation and inflammatory astrocytopathy mediated by these antibodies, while AQP4 ab− patients often exhibit distinct immunological profiles. Growing evidence indicates that the interaction between specific cytokine profiles and AQP4 expression is critical for differentiating NMOSD subtypes, affecting disease activity and local immune responses. Exposure of astrocytes to cytokines such as IL-1β, IL-6, IL-10 and IL-17A significantly alters AQP4 expression and complement activation, highlighting the critical role of these cytokines in distinguishing immunological differences between AQP4 ab+ and ab- patients [7].

Furthermore, molecules involved in extracellular matrix remodeling and angiogenesis, such as Thrombospondin-1 and Vascular Endothelial Growth Factor (VEGF), have emerged as important contributors to the neuroinflammatory environment and tissue repair processes in NMOSD. Their profiles potentially reflect disease state and immune environment variations, offering additional avenues for diagnostic and monitoring biomarker development [8,9]. In the recent scenario of approved NMOSD treatments targeted to monoclonal antibodies, diagnostic and monitoring biomarkers emerge as power tools in decision-making strategies.

Here, we aimed to investigate the role of cytokines and growth factors associated with astrocyte function and response to injury as NMOSD biomarkers. Further, we evaluated the ability of these molecules to discriminate between AQP4 ab+ and AQP4 ab− NMOSD profiles and other IDDs. Our data showed an association between the plasmatic concentrations of transforming growth factor β (TGF-β) and AQP4 ab status and disability in NMOSD patients. Together, our data show a potential role for TGF-β as a biomarker in NMOSD astrocytopathy.

## 2. Materials and Methods

### 2.1. Clinical Data Review

Patients (176) were included from the setting of an IDDs reference center at the Clementino Fraga Filho University Hospital, a tertiary hospital in Rio de Janeiro, Brazil. The timeframe of the medical records comprised a period between the years of 1976 and 2022. Data regarding time of onset, disease severity, committed functional systems, time between relapses, MRI lesion content, follow-up time, disease-modifying therapies and employed diagnostic tools were compiled. Annualized relapse rate (ARR) was calculated as a ratio between total number of relapses and total follow-up time in years. The expanded disability scale score (EDSS) and functional systems were discriminated as firstly proposed by John Kurtzke [10] and applied to define severity outcomes phenotypes. For NMOSD, the 2015 international consensus diagnostic criteria [11] were considered; for MS and RIS, the 2024 revisions of the McDonald criteria [12]; for MOGAD, the international MOGAD panel proposed criteria from 2023 were adopted [13]; and for ADEM, diagnoses were performed according to previous reports [14].

For details regarding the study design, please refer to the STROBE Statement-Checklist (Table S1).

### 2.2. Sample Selection

An anamnesis, physical examination and evaluation of an MRI within the previous six months were performed to confirm disease status. Only patients with an established diagnosis and a follow-up time equal or longer than one year were included. Patients which sported other neurological, psychiatric or autoimmune diseases, those with ongoing infections or with other causes of disability were excluded. For controls, volunteers between 18 and 70 years old, only anamnesis and physical examination were executed.

### 2.3. Sample Collection

Blood samples were collected from all subjects, using EDTA and serum-separating gel tubes to obtain plasma, serum and buffy coat. Samples were centrifuged at 750× *g* for 10–15 min at room temperature to separate components. Plasma and serum aliquots were stored at −20 °C to prevent contamination, and all samples were subsequently kept at −80 °C for long-term storage in a designated LABNET freezer, with locations logged systematically.

### 2.4. Interleukins and Growth Factors Measurement

TGF-β concentration was determined using the Simoa^®^ TGF-β Discovery Kit (Quanterix Corp., Billerica, MA, USA; Cat#101984) for the HD-1 analyzer, adapted to the SR-X system as a 3-step protocol, according to instructions provided by the manufacturer in the Quanterix Assay Conversion Guide: HD-1 to SR-X. Calibrator curves were performed as triplicates, while samples were tested as duplicates.

Concentrations of interleukins-1β (IL-1β), -8 (IL-8), -10 (IL-10) and 17-A (IL-17A) were accessed through a multiplex assay, using the Essential 4-Plex Human ProcartaPlex™ Panel 2 kit (Thermo Fisher Scientific Inc., Waltham, MA, USA; Cat# EPX040-10008-901) according to the manufacturer’s instructions, and readings were performed on a Luminex 200™ System (Merck KGaA, Darmstadt, Germany). Concentrations of Thrombospondin-1 and Vascular Endothelial Growth Factor (VEGF) were accessed through sandwich ELISA kits (R&D Systems, Inc., Minneapolis, MN, USA; Cat# DVE00 and DTSP10). Colorimetric readings were performed in a spectrophotometer at wavelengths of 450 nm, with a correction set at 570 nm. Analyte concentrations were calculated according to the standard curves of each assay. All tests were performed in duplicates.

### 2.5. Human Astrocyte Culture and Treatments

Adult primary human astrocytes were isolated from surgically removed anterior temporal lobe tissue from patients selected for surgical treatment of temporal lobe epilepsy associated with hippocampal sclerosis (TLE-HS), as previously described [15]. Confluent astrocyte cultures were incubated with Dulbecco’s minimum essential medium and nutrients mixture F12 (DMEM/F12, Invitrogen), without FBS for 18–24 h prior to the treatment, with 5% human serum derived from AQP4 ab-positive and AQP4 ab-negative NMOSD patients or from AQP4 ab-positive subjects supplemented with 10 ng/mL recombinant TGF-β1 for 24 h.

### 2.6. Immunocytochemistry and Densitometric Analysis

Astrocyte cultures were fixed with 4% paraformaldehyde (PFA), and immunoassays were performed according to Matias et al. [16] Primary antibodies were rabbit anti-GLT-1 (1:100; Abcam, Cambridge, UK), mouse anti-glutamine synthetase (1:100; Millipore, Burlington, MA, USA), rabbit anti-GLAST (1:100; Abcam, Cambridge, UK), rabbit anti-C3 (1:100, Abcam, Cambridge, UK), mouse anti-HMGB1 (1:100, Invitrogen, Carlsbad, CA, USA) and rabbit anti-NFkB p65 (1:100, Abcam, Cambridge, UK). Secondary antibodies were Alexa Fluor 488-conjugated goat anti-rabbit IgG or goat anti-mouse IgG (1:300; Invitrogen, Carlsbad, CA, USA), or Alexa Fluor 546-conjugated goat anti-rabbit IgG or goat anti-mouse IgG (1:1000; Invitrogen, Carlsbad, CA, USA). Nuclei were counterstained with DAPI (Sigma-Aldrich, St. Louis, MO, USA), and cells were observed with a TE2000 Nikon microscope. Densitometry for the images was performed using integrated density values generated by ImageJ software version 1.53q (NIH) and normalized by the number of cells per field. The analysis of HMGB1 nucleus/cytoplasm ratio was adapted from Noursadeghi et al. [17] to quantify HMGB1 cytoplasmic translocation. For each experimental condition, images were acquired from three independent cultures: 50 images for HMGB1, 50 for NFkB, 63 for C3, 83 for GS, 63 for GLAST and 81 for GLT-1. These images corresponded to approximately 10–40 fields per culture, depending on the marker.

### 2.7. Statistical Analysis

Statistical analysis and graphical representation were executed using GraphPad Prism software version 10.0.1 (GraphPad Software, La Jolla, CA, USA). Student’s *t*-test was used when comparison between two groups was needed. For data regarding multiple groups, analysis of variance (ANOVA) was used. Nondirectional Tukey’s post-test was performed when statistical significance was determined. Survival analysis was calculated as a percentage over time. Difference between survival curves was determined by a Mantel–Cox test. Correlation analysis was performed between two different parameters through simple linear regression. The confidence interval used was 95%, considering statistical significance a *p*-value under 0.05. Data was expressed on graphs as the mean ± SEM, the error bars in the graphs being the SEM. Individual dots or marks on graphs represent an individual experimental subject. All tests were performed in replicates, with the mean of replicates for a similar subject considered as the subject’s value for data analysis.

## 3. Results

### 3.1. Onset Characteristics of Inflammatory Demyelinating Diseases at a Tertiary Hospital

**Figure 1:**
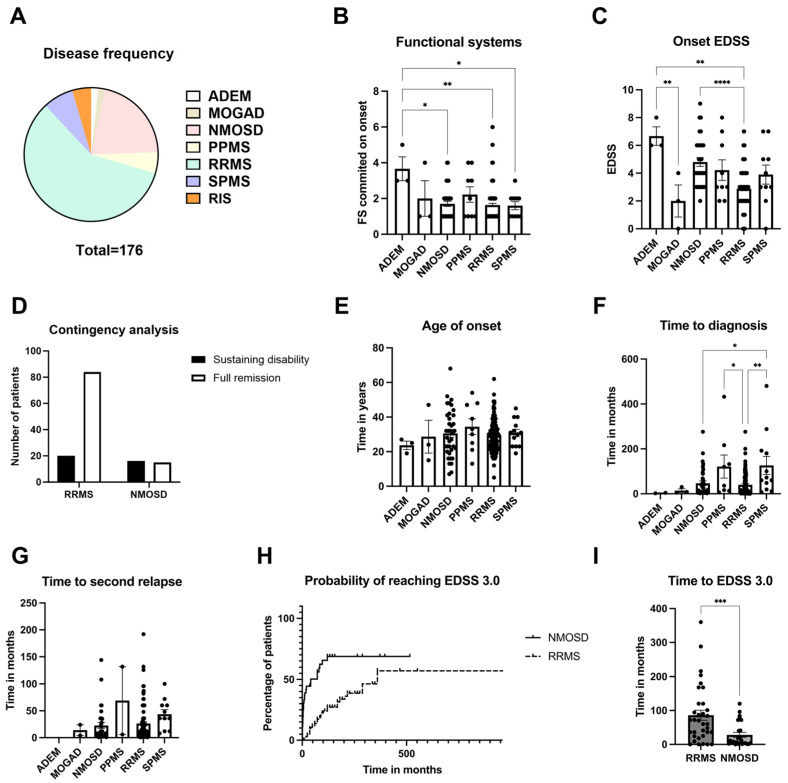
Clinical characteristics of a CNS demyelinating diseases cohort in a tertiary hospital. Parts of the whole graph demonstrate disease distribution among the cohort (**A**). The largest groups were composed of RRMS and NMOSD patients. Next, bar graphs depict the disease burden sustained on onset, as evaluated by both number of affected function systems (**B**) and highest EDSS achieved at the time (**C**). The highest EDSS values were found for ADEM and NMOSD patients. Contingency analysis showed that, among the largest groups, NMOSD patients were also more likely to sustain disability after the first attack than RRMS ones (**D**). Age of disease onset in years (**E**). Time taken between onset and diagnosis was measured in months and depicted as a bar graph (**F**). Time elapsed between onset and the next relapse was also measured in months (**G**). Survival analysis showed that NMOSD patients were more likely to reach EDSS 3.0 than RRMS patients. Horizontal axis represents time in months, while vertical axis represents a percentage of patients in the disease group (**H**). Analysis demonstrated in the bar graphs also showed that NMOSD patients reached EDSS 3.0 in a shorter time than those with RRMS (**I**). Bar graphs depict the means +/− SEM (*n* ≥ 3), and each dot represents an individual subject. * *p* < 0.05, ** *p* < 0.01, *** *p* < 0.001, **** *p* < 0.0001.

Among the total of 176 IDDs patients included with current follow-up, groups were classified with the following diagnoses: ADEM (3); MOGAD (3); NMOSD (37); primary progressive multiple sclerosis (PPMS) (9); relapsing-remitting multiple sclerosis (RRMS) (103); secondary progressive multiple sclerosis (SPMS) (13); and 8 fulfill criteria of radiologic isolated syndrome (RIS) (Figure 1A). Demographic characteristics are further described in Table 1.

A tendency was found for patients with PPMS to start with pyramidal symptoms when compared to others, but correlation analysis did not find it statistically significant. Patients with ADEM had both the highest number of affected FS (Figure 1B; mean 3.67) and the highest EDSS (Figure 1C; mean 6.67). Other diseases did not differ in the number of affected FS. Finally, NMOSD patients had a higher EDSS than RRMS ones (Figure 1C; means 4.60 and 2.88, respectively).

NMOSD patients were also less likely to have full remission after their first event than those with RRMS (Figure 1D). Age of onset between groups did not vary (Figure 1E). Independent of the disease, the time to diagnosis was similar (Figure 1F). There was no difference in the meantime to a second relapse either (Figure 1G). Concerning permanent disability, NMOSD patients also showed a higher probability of reaching EDSS 3.0 than RRMS ones (Figure 1H), while also achieving it in a shorter period (Figure 1I; mean 28.4 months for NMOSD; 86.0 months for RRMS).

### 3.2. Characteristics of NMOSD Patients According to AQP4 ab Status

**Figure 2:**
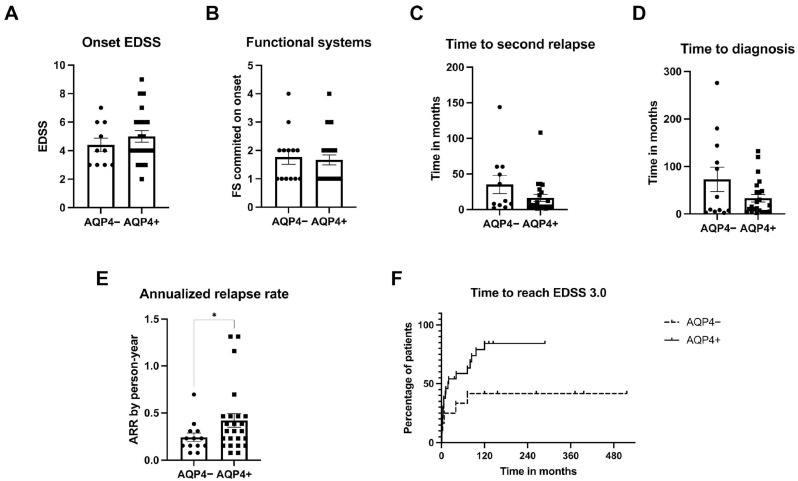
NMOSD patients presented different clinical outcomes according to AQP4ab serum positivity. All graphs show clinical parameters of NMOSD patients, stratified according to negativity (AQP4−) or positivity (AQP4+) for AQP4ab in a cell-based assay. The first four graphs evaluate onset characteristics. A bar graph compares the highest EDSS achieved during the first attack (**A**). The next one shows the number of functional systems impaired at the time (**B**). The third bar graph depicts the time elapsed in months between the first and second attacks (**C**). The fourth bar graph shows the time in months between the onset of symptoms and the diagnosis (**D**). AQP4ab presence did not affect any of those parameters in the cohort, which is divergent to what was seen in follow-up. A bar graph shows both groups’ annualized relapse rate calculated as a person–years variable (**E**). A graph demonstrates the time taken by patients to reach an EDSS of 3.0 as a survival rate analysis. The horizontal axis depicts time in months and the vertical axis the percentage of patients within the group to achieve the endpoint. Each line represents a group (**F**). Observe that the AQP4+ group presents both the higher ARR and the higher probability of achieving EDSS 3.0. The bar graphs depict the means +/− SEM (*n* ≥ 9). Dots represent individual subjects. * *p* < 0.05.

Clinical evaluations comparing AQP4 ab+ and AQP4 ab− NMOSD groups were performed. Both showed similar onset characteristics. There were no differences on the disability reached according to the EDSS during the first attack (means 4.4 and 5.0, respectively; Figure 2A), as well as in the number of committed FS at the time (means 1.77 and 1.67; Figure 2B) or in the time taken between the first and second attacks (means 35.27 and 16.41; Figure 2C). The presence or not of the AQP4 ab also did not impact the time in months needed to reach the NMOSD diagnosis (means 72.75 and 33.26; Figure 2D).

Moreover, while both groups were under treatment and showed similarity in the initial phases of disease, AQP4 ab+ patients presented a higher annualized relapse rate (ARR; mean 0.42) than the AQP4 ab− group (mean 0.24; Figure 2E). The results revealed that not only a greater percentage of AQP4ab+ patients reached EDSS 3.0 during disease course (84.29%) than of the AQP4 ab− subjects (41.67%) but also accomplished that within a shorter timeframe (Figure 2F).

### 3.3. Plasmatic Concentrations of Interleukins and Growth Factors in NMOSD

**Figure 3:**
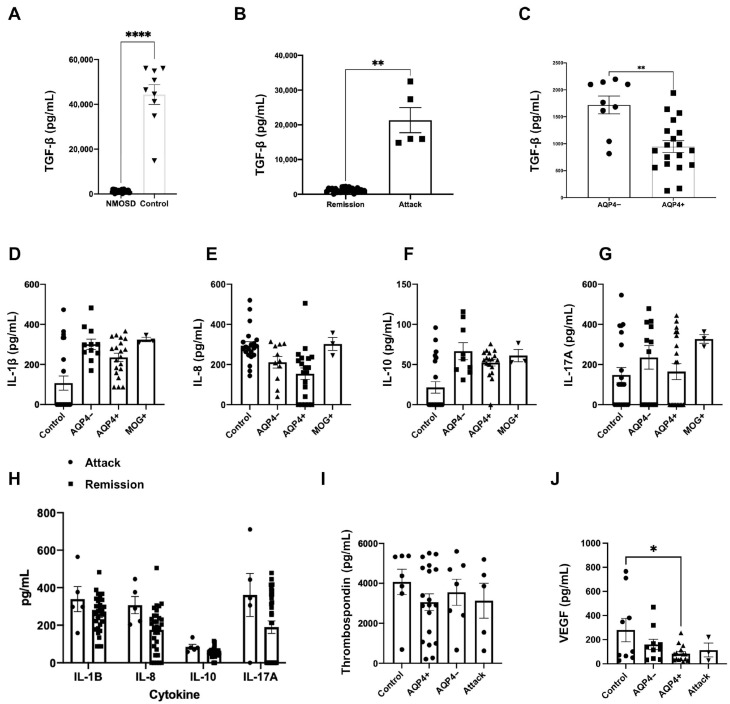
Expression of TGF-β in NMOSD patients is dependent on antibody status. All images are bar graphs representing concentrations of analytes in plasma as pg/mL. The first three graphs represent TGF-β concentrations in plasma of NMOSD patients measured through SIMOA assays. TGF-β concentrations are reduced in NMOSD patients when compared to controls (**A**). In the next graph, it can be seen that those values increase during NMOSD active phase (**B**). Lastly, it is observed that, when inactive NMOSD patients are stratified according to serum positivity for AQP4ab, AQP4ab+ subjects show the lowest titles of TGF-β (**C**). The next five graphs show dosages of interleukins performed with a Luminex assay. Interleukin 1B (IL-1β) (**D**), interleukin 8 (IL-8) (**E**), interleukin 10 (IL-10) (**F**) and interleukin 17A (IL-17A) (**G**) were measured in healthy controls. NMOSD patients stratified according to negativity (AQP4−) or positivity (AQP4+) to AQP4ab and MOGAD patients (MOG+). No difference was seen between groups. Interleukin levels were also compared between NMOSD patients according to disease activity (**H**), also with no variance. The growth factors Thrombospondin and Vascular Endothelial Growth Factor (VEGF) were evaluated through ELISA. Columns represent healthy controls; NMOSD patients with inactive disease stratified by AQP4ab and NMOSD patients with ongoing attack. Thrombospondin did not vary between groups (**I**). VEGF was slightly reduced in the AQP4+ group, but only when compared to controls (**J**) (Bar graphs depict the means +/− SEM (*n* ≥ 3). * *p* < 0.05, ** *p* < 0.01, **** *p* < 0.0001).

The concentration of TGF-β in NMOSD plasma was lower than in healthy controls (mean 1195.14 pg/mL; Figure 3A). Conversely, during an NMOSD attack, the concentrations became 17.84-fold higher than in a controlled disease scenario (Figure 3B) but was still lower than healthy controls. Furthermore, among NMOSD patients with controlled disease, the TGF-β titles were even lower in patients with AQP4 ab serum-positive disease (mean 947.529 pg/mL; Figure 3C) than in serum-negative NMOSD (mean 1718.22 pg/mL).

There was no significant difference in interleukins’ (IL-1B, IL-8, IL-10, IL-17a) plasma concentrations between AQP4 ab− and AQP4 ab+ (Figure 3D–G). The levels were comparable between those seen in healthy controls and in MOGAD patients. Disease activity also had no impact on those concentrations (Figure 3H).

Thrombospondin concentrations did not vary between groups (Figure 3I), and VEGF was slightly reduced in AQP4 ab+ patients (mean 84.112 pg/mL) when compared to healthy controls (mean 279.983 pg/mL; Figure 3J). This difference was suppressed in the group of patients suffering from a disease attack (mean 114.112 pg/mL).

**Figure 4:**
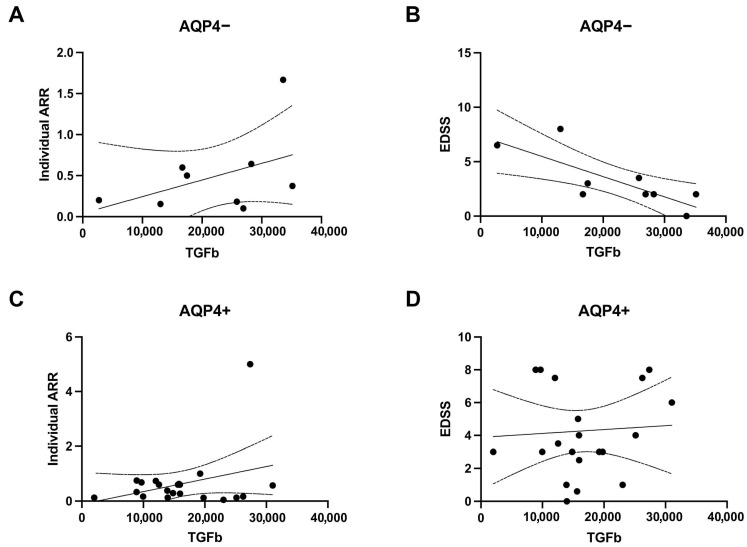
TGF-β correlation with clinical outcomes in NMOSD patients. All graphs represent a correlation analysis between plasmatic TGF-β and disease burden parameters. Horizontal axis in all graphs depict TGF-β concentration in pg/mL. (**A**,**B**) AQP4 ab serum-negative group (AQP4−) and (**C**,**D**) AQP4 ab serum-positive group (AQP4+). Vertical axes represent the individual annualized relapse rate (Individual ARR) (**A**,**C**) and EDSS at the time of sample collection (**B**,**D**). The only regression with a significant correlation was of EDSS in the AQP4− group. Observe how higher titles of TGF-β coincide with lower EDSS values. The straight lines represent the expected function obtained through linear regression. Area between parables depicts the 95% CI. Each dot on the graph represents an individual subject.

Linear regressions between TGF-β titles and ARR or EDSS for both AQP4 ab− (Figure 4A,B) and AQP4 ab+ (Figure 4C,D) groups were calculated. The EDSS presented a significant correlation with TGF-β when considering the AQP4 ab− patients (Figure 4B). Higher EDSS values were found to correlate with lower TGF-β concentrations, a finding which was specific to this group.

### 3.4. TGF-β Plasmatic Concentrations Among Different Disease Groups

**Figure 5:**
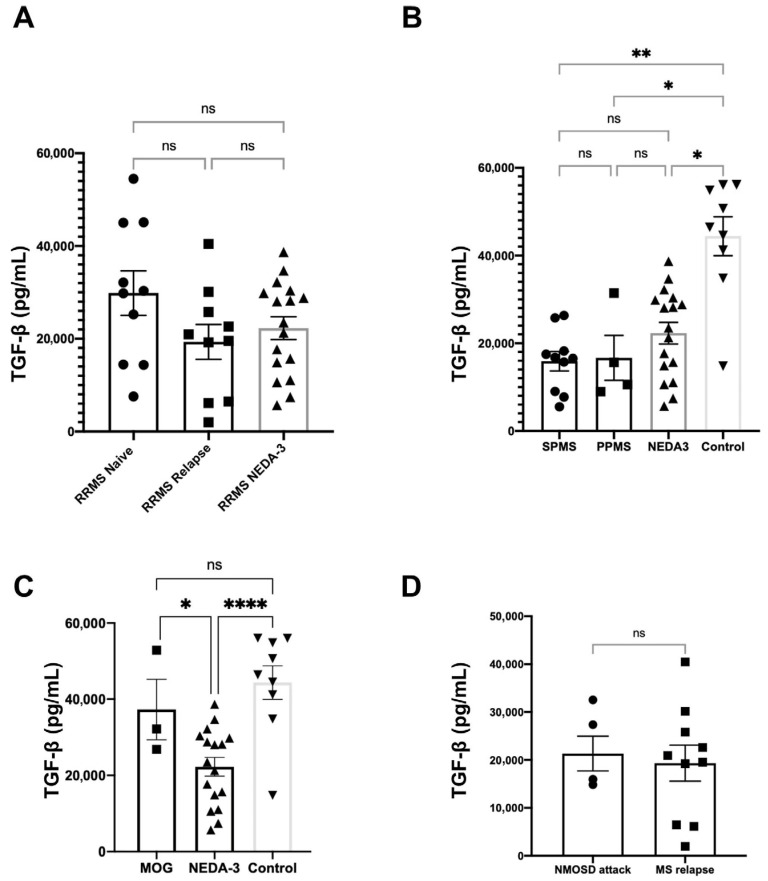
TGF-β is differentially expressed according to CNS IDDs. All graphs represent TGF-β concentrations in plasma measured through SIMOA assays in pg/mL. Bar graph represents TGF-β titles in different phases of RRMS. Columns depict asymptomatic patients naïve to treatment, treated patients on relapse at the time of assay and treated patients with controlled disease by NEDA-3 criteria (**A**). No variation was seen. Comparisons were also performed regarding disease progression for MS. Bar graph shows concentrations for SPMS, PPMS and NEDA-3 RRMS patients. A fourth column depicts healthy controls (**B**). Note there is no variation in TGF-β between MS phenotypes, but all of them remain below control levels. The next bar graph compares NEDA-3 RRMS subjects with inactive MOGAD individuals, as well as healthy controls (**C**). While RRMS shows lower concentrations than the other groups, MOGAD presents titles close to those of healthy controls. A graph compares NMOSD and RRMS TGF-β levels during diseases’ active phases (**D**), with no difference between them. The bar graphs depict the means +/− SEM (*n* ≥ 3), and each dot represents an individual subject. * *p* < 0.05, ** *p* < 0.01, **** *p* < 0.0001, ns *p* ≥ 0.05.

Concerning RRMS subjects, no difference was seen between those naïve to treatment (mean 28,844.7 pg/mL), those on a relapse (mean 19,323.9 pg/mL) and those with a controlled disease according to NEDA-3 criteria (mean 25,898.2 pg/mL; Figure 5A). TGF-β levels of RRMS NEDA-3 were also like those of treated SPMS (mean 15,902.7 pg/mL) and PPMS (mean 16,658.3 pg/mL). All groups showed a lower concentration than the healthy controls (mean 44,388.3 pg/mL; Figure 5B).

MOGAD subjects had titles higher than MS groups and close to controls (mean 37,334.7 pg/mL; Figure 5C). All MOGAD patients were under treatment at the time of the tests and were in remission. When comparing all disease groups, NMOSD patients presented levels much lower than both MOGAD and NEDA-3 subjects. Concentrations found in RRM relapse (19,323.9 pg/mL) and NMOSD attack (21,320.5 pg/mL) phases were similar (Figure 5D).

### 3.5. Expression of Inflammation and Glutamate Metabolism Markers on Cultured Human Astrocytes Treated with NMOSD Patient Serum

**Figure 6:**
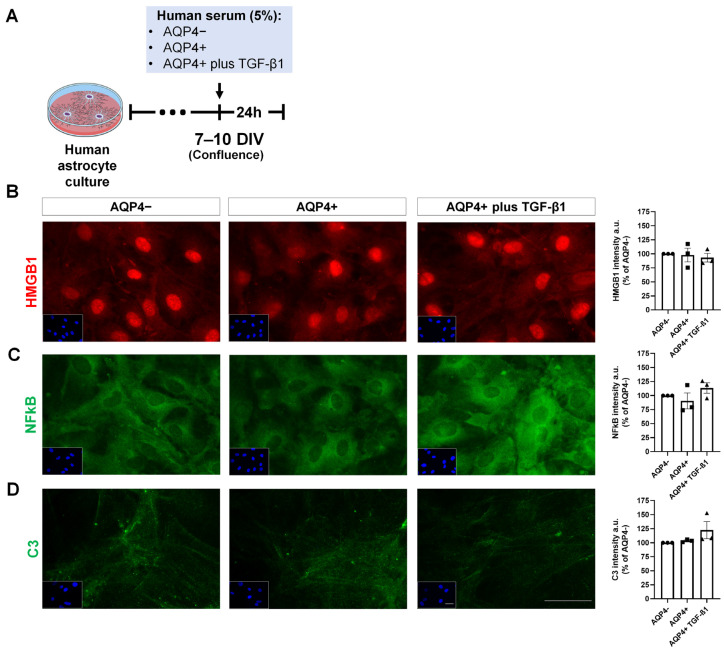
Inflammatory response of astrocytes induced by NMOSD subjects’ serum does not correlate to AQP4 ab serum positivity. Schematic representation of the in vitro experimental design (**A**). Human astrocytes cultures were treated with serum either from an AQP4ab NMOSD subject (AQP4−), from an AQP4ab+ NMOSD subject (AQP4+) or from an AQP4ab+ subject supplemented with recombinant TGF-β1 (AQP4+ TGF-β1). After treatment, cultures were labeled by immunofluorescence for proteins associated with astrocyte reactivity. Insets show blue labeling of nuclei with DAPI. Cells were labeled for HMGB1 (red), (**B**), NFkB (green), (**C**) and C3 (green), (**D**). No difference was found for those markers between groups. All bar graphs represent the mean integrated density of labeling for the images of each group expressed as a percentage of the mean for AQP4−. Each dot is an experimental unit. Bar graphs depict means +/− SEM. Scale bars: 40 µm.

The correlation between astrocyte reactivity and function and AQP4 ab serological status was investigated. Human astrocytes were cultured in the presence of serum from an AQP4 ab− NMOSD patient (AQP4−) or an AQP4ab+ patient (AQP4+) followed by immunofluorescence for markers of astrocyte reactivity and glutamate metabolism. Exposure of astrocytes to AQP4 ab+ NMOSD patient serum (and TGF-β1) did not impact any of the inflammatory molecules tested: HMGB1, NFkB, C3 and LCN2 (Figure 6).

**Figure 7:**
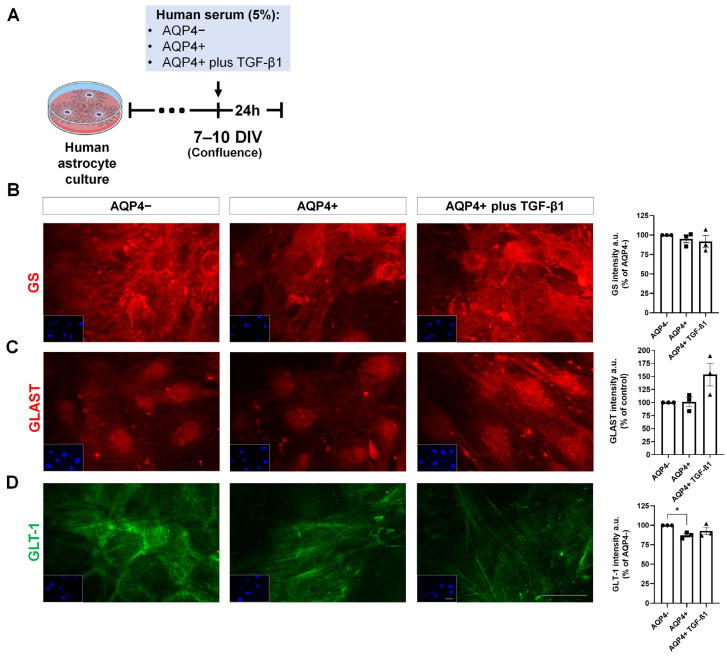
GLT-1 expression in astrocytes is impaired by AQP4 ab+ NMOSD serum. Schematic representation of the in vitro experimental design (**A**). Human astrocyte cultures were treated with serum either from an AQP4ab- NMOSD subject (AQP4−), from an AQP4ab+ NMOSD subject (AQP4+), or from an AQP4ab subject supplemented with recombinant TGF-β1 (AQP4+TGF-β1). After treatment, cultures were labeled by immunofluorescence for proteins associated with glutamate metabolism. Insets show blue labeling of nuclei with DAPI. Cells were labeled for glutamine synthetase (GS) (red), (**B**), GLAST (red), (**C**) and GLT-1 (green), (**D**). Of note, a reduction in GLT-1 intensity can be seen in the AQP4+ group, which is rescued by TGF-β1 supplementation. All bar graphs represent the mean integrated density of labeling for the images of each group expressed as a percentage of the mean for AQP4−. Each dot is an experimental unit. Bar graphs depict means +/− SEM. Scale bars: 40 µm. * *p* < 0.05.

Since glutamatergic excitotoxicity acts as a mechanism of gray matter disease in IDDs [18], the staining of molecules involved in glutamate metabolism was evaluated. GS (glutamine synthetase) and glutamate-aspartate transporter (GLAST) showed no variation among the different treatments (Figure 7B,C). Treatment of astrocytes with AQP4 ab+ serum triggered a slight reduction in GLT-1 levels when compared to the mean of the AQP4 ab− group (mean 87.13% of AQP4−; Figure 7C). The addition of recombinant TGF-β1 to AQP4 ab+ serum suppressed this effect (mean 92.80% of AQP4−; Figure 7C).

## 4. Discussion

Our results show experimental evidence of TGF-β as a potential biomarker for NMOSD and contribute to understanding the mechanisms underlying AQP4 ab seronegative and seropositive groups of this disease.

Although IDD cases showed a high overlapping clinical profile between those diseases on onset, NMOSD patients presented poorer clinical outcomes, as previously shown by our group [19]. They had a higher chance to evolve with disability and reach it at faster times and higher rates than RRMS subjects, even though the RRMS group presented a higher ARR. While both groups had high adherence to disease-modifying drug (DMD) use, NMOSD patients in Brazil were on off-label treatments, mostly with azathioprine and rituximab. As specifically designed NMOSD therapy has been approved [20]; this panorama may change in the near future.

Regardless of severity, when investigating plasmatic TGF-β content in IDDs, all groups showed smaller concentrations of this cytokine than controls. A study with RRMS patients already showed decreased TGF-β mRNA expression in CD4+T cells when compared to a control group [21]. This was accompanied by higher IL-6 expression, where it seemed to normalize with interferon treatment. Another cross-sectional study showed reduction in TGF-β titles in serum of RRMS and clinically isolated syndrome (CIS) patients [22].

TGF-β1 upregulation was reported in NMOSD patients when compared to MS patients and patients with non-inflammatory neurological disease [23]. This report focused on newly onset untreated subjects and lacked comparison to healthy controls. NMOSD subjects were stratified according to AQP4 ab serum positivity, but no difference was observed. On the contrary, our data shows that total TGF-β plasma concentrations are significantly different between AQP4 ab− and AQP4 ab+ patients, with serum-positive patients presenting the lowest titles. During attacks, the concentrations of TGF-β in NMOSD increased up to the ones normally seen in MS patients. This may be due to an adaptive response to the inflammatory insult, rather than a deleterious component of it. TGF-β decrease has also been reported in the in vivo EAE model [24] as well as in in vitro exposition of astrocytes to myelin antigens, typically seen in IDDs [25].

In CNS, TGF-β1 production by astrocytes is associated with neuronal survival and synapse formation [15,26,27] as well as with several neurodegenerative diseases [28,29,30].

TGF-β concentrations were shown to be inversely proportional to the EDSS at the time of testing. Although the EDSS can be considered a non-linear measure and may be criticized for undervaluing non-motor symptoms, this is the first case scenario where plasmatic laboratory testing can represent a clinical parameter specifically for AQP4 ab− NMOSD. Prospective studies are still needed to evaluate if the correlation remains in larger populations and if it holds a prognostic value.

Previously, different strategies have been tested to determine prognosis in NMOSD, but they could not be applied to AQP4 ab− subjects [31]. In our cohort, the absence of AQP4 ab did not delay diagnosis or treatment in NMOSD patients. While having similar onsets, AQP4 ab+ patients had a higher annualized relapse rate despite treatment and had higher chances of achieving a higher EDSS, for which there was a tendency to happen in shorter timeframes. Our data is supported by a recent case series in China, which demonstrated a difference of 1.52 between EDSS means of AQP4 ab+ and AQP4 ab− NMOSD groups [32]. Interestingly, our AQP4 ab− group, which can be considered the one clinically less severe in our cohort, had the highest concentrations of TGF-β.

The lack of proper understanding of cellular mechanisms underlying AQP4 ab+ and AQP4 ab− disease led us to investigate if the differences found between factors in AQP4 ab+ and AQP4 ab− subjects could be explained by direct action of AQP4 ab on astrocytes. To address this, in vitro assays with human astrocytes treated with serum from NMOSD patients of both serotypes were performed. The binding of the antibody to AQP4 is reported to directly impact cellular functions such as induction of IL-6 release, internalization of GLT-1 [33] and induction of ATP release [34]. Interestingly, midbrain astrocytes from knockout mice for AQP4 fail to produce TGF-β [35].

Treatment of human astrocytes with serum from different serotype patients did not impact labeling for inflammatory markers, independent of the serotype exposed. Therefore, it would be unlikely for TGF-β1 to exert modulatory effects on these markers under our experimental conditions, as no baseline increase was observed. Nonetheless, a reduced density of GLT-1 labeling on the cell surface was seen when astrocytes were exposed to serum from an AQP4 ab+ patient, an event rescued by TGF-β treatment, suggesting that TGF-β1 preferentially targeted glutamate metabolism rather than inflammatory cascades in this in vitro context. Although the ability of TGF-β1 to protect astroglia against glutamatergic metabolism impairment has not been investigated in NMOSD, this has been established in other disease contexts. Our group has shown that exposure of astrocytes to α-synuclein oligomers increased GLAST and GLT-1 expression and that pharmacological inhibition of TGF-β1 signaling in vivo prevented this upregulation, highlighting the involvement of TGF-β in glutamate transporter regulation in a mouse model of synucleinopathy [33]. This notion is further supported by [36] who demonstrated that mice lacking TGF-β1 in the CNS display impaired glutamate recycling due to reduced GLT-1 and GLAST expression. Moreover, astrocyte cultures treated with TGF-β1 exhibited increased GLAST and GLT-1 expression, as well as enhanced glutamate uptake [36]. Therefore, the effects of TGF-β on astrocytes are highly context-dependent [37], with consistent evidence linking this pathway to protection against glutamate-mediated excitotoxicity through the upregulation of glutamate transporters [36,38].

Our results confirm a mechanism of cell dysfunction in NMOSD to be exclusive for AQP4 ab+ disease. This further suggests that seronegative cases can represent a separate nosological entity. This understanding is essential for biomarker discovery and DMD development. Emerging therapies for NMOSD have no conclusive roles in AQP4 ab− disease. We understand that further characterization of biomarkers and biological processes specific to the AQP4 ab− NMOSD forms will be beneficial in the future, as it may help to define follow-up strategies and guide research for therapeutic targets.

## 5. Conclusions

Here, we showed evidence for the use of plasmatic TGF-β as a biomarker candidate for demyelinating diseases, especially in the context of NMOSD. In this disease group, greater variations in TGF-β were seen, along with a novel clinical correlation exclusive to AQP4 ab− NMOSD. Additionally, TGF-β levels may represent a potential biomarker to monitor therapeutic response in NMOSD, although further studies to clarify how TGF-β modulation could influence treatment strategies in AQP4 antibody-positive patients are needed. Reduced labeling of glutamate transporters in AQP4 ab+ cultures represent further evidence for differing pathological mechanisms between the two forms of NMOSD. We understand that further characterization of biomarkers and biological processes specific to the AQP4 ab− NMOSD forms, as well as an increase in the number of patients, will be beneficial in the future, as it will help to better define follow-up strategies and may guide research for therapeutic targets.

## Figures and Tables

**Table 1:** Demography of CNS IDDs patients in a tertiary hospital cohort.

	ADEM	MOGAD	NMOSD	PPMS	RRMS	SPMS	RIS
**Number of patients**	3 (1.70%)	3 (1.70%)	37 (21.02%)	9 (5.11%)	103 (58.52%)	13 (7.39%)	8 (4.54%)
**Gender** **(% of total)**	F: 66.67% M: 33.33%	F: 33.33% M: 66.67%	F: 86.49% M: 13.51%	F: 33.33% M: 66.67%	F: 71.85% M: 28.15%	F: 76.92% M: 23.08%	F: 62.50% M: 37.50%
**Age** **(Years)**	27.33 ± 4.33	35.00 ± 9.61	43.49 ± 2.44	52.78 ± 4.88	42.25 ± 1.06	55.15 ± 3.08	44.88 ± 7.18
**Scholarity** **(% of total)**	PS: 0.00% MS: 0.00% HS: 100.00% HE: 0.00%	PS: 0.00% MS: 33.33% HS: 66.67% HE: 0.00%	PS: 11.11% MS: 33.33% HS: 44.44% HE: 11.11%	PS: 0.00% MS: 50.00% HS: 33.33% HE: 16.67%	PS: 5.43% MS: 9.78% HS: 43.48% HE: 41.30%	PS: 11.11% MS: 11.11% HS: 55.55% HE: 22.22%	PS: 12.50% MS: 25.00% HS: 37.50% HE: 25.00%
**Race** **(% of total)**	B: 0.00% MR: 33.33% W: 33.33% NI: 33.33%	B: 0.00% MR: 0.00% W: 100.00% NI: 0.00%	B: 16.22% MR: 27.03% W: 45.95% NI: 10.81%	B: 22.22% MR: 33.33% W: 44.44% NI: 0.00%	B: 1.00% MR: 23.30% W: 64.08% NI: 11.65%	B: 0.00% MR: 23.08% W: 61.54% NI: 15.38%	B: 0.00% MR: 0.00% W: 75.00% NI: 25.00%
**Follow-up time (Years)**	3.67 ± 2.19	6.33 ± 1.20	12.95 ± 1.79	18.33 ± 4.12	13.31 ± 0.83	24.38 ± 3.35	8.86 ± 3.14
**ARR (Person-years)**	0.27	0.37	0.36	0.09	0.45	0.39	0.24
**DMD use (% of total)**	0.00%	100.00%	89.18%	55.55%	97.11%	76.93%	25.00%

Abbreviations: F: female; M: male; PS: primary school; MS: middle school; HS: high school; HE: higher education; B: black; MR: mixed race; W: white; NI: no racial identification; ARR: annualized relapse rate; RIS: radiologic isolated syndrome; DMD: disease-modifying drug.

## Data Availability

The original contributions presented in this study are included in the article/Supplementary Material. Further inquiries can be directed to the corresponding authors.

## Supplementary Materials

The following supporting information can be downloaded at: https://www.mdpi.com/article/10.3390/neurolint17120200/s1, Table S1: STROBE Statement.
